# Discovery of novel cardiac troponin activators using fluorescence polarization-based high throughput screening assays

**DOI:** 10.1038/s41598-023-32476-w

**Published:** 2023-03-30

**Authors:** Priyanka Parijat, Saraswathi Ponnam, Seetharamaiah Attili, Kenneth S. Campbell, Mohammed El-Mezgueldi, Mark Pfuhl, Thomas Kampourakis

**Affiliations:** 1 Randall Centre for Cell and Molecular Biophysics, King’s College London, London, SE1 1UL UK; 2 British Heart Foundation Centre of Research Excellence, King’s College London, London, SE1 1UL UK; 3 Division of Cardiovascular Medicine and Department of Physiology, University of Kentucky, Lexington, KY USA

**Keywords:** Biochemistry, Proteins, Contractile proteins, Drug discovery, Drug screening, High-throughput screening

## Abstract

The large unmet demand for new heart failure therapeutics is widely acknowledged. Over the last decades the contractile myofilaments themselves have emerged as an attractive target for the development of new therapeutics for both systolic and diastolic heart failure. However, the clinical use of myofilament-directed drugs has been limited, and further progress has been hampered by incomplete understanding of myofilament function on the molecular level and screening technologies for small molecules that accurately reproduce this function in vitro. In this study we have designed, validated and characterized new high throughput screening platforms for small molecule effectors targeting the interactions between the troponin C and troponin I subunits of the cardiac troponin complex. Fluorescence polarization-based assays were used to screen commercially available compound libraries, and hits were validated using secondary screens and orthogonal assays. Hit compound-troponin interactions were characterized using isothermal titration calorimetry and NMR spectroscopy. We identified NS5806 as novel calcium sensitizer that stabilizes active troponin. In good agreement, NS5806 greatly increased the calcium sensitivity and maximal isometric force of demembranated human donor myocardium. Our results suggest that sarcomeric protein-directed screening platforms are suitable for the development of compounds that modulate cardiac myofilament function.

## Introduction

Heart failure and heart disease remain the predominant causes for death world-wide and are a significant burden for both the healthcare systems and global economies^1,2^. Current heart failure therapies are primarily aimed at symptomatic relief via neurohumoral modulation, but usually do not treat the underlying etiologies of heart failure and can cause unwanted side-effects^3^. For example, although commonly used inotropes such as dobutamine increase cardiac output, treatment is usually associated with severe side effects such as arrhythmias and hypotension^4,5^. Consequently, there is a largely unmet demand for new pharmacological therapies for heart disease and heart failure.

It has become increasingly evident that cardiac myofilament proteins are important regulators of heart muscle function during both health and disease states. This is highlighted by the fact that mutations in the genes encoding for the molecular motor β-cardiac myosin, the cardiac isoform of myosin binding protein-C, the cardiac troponin subunits and tropomyosin are found in the majority of patients suffering from inherited Hypertrophic Cardiomyopathy (HCM)^6^. Additionally, post-translational modifications of these sarcomeric proteins are important regulators of both systolic and diastolic function^7,8^, and protein hypo-phosphorylation has been frequently associated with heart muscle dysfunction and heart failure in both human patients and animal models^9,10^.

**Figure 1:**
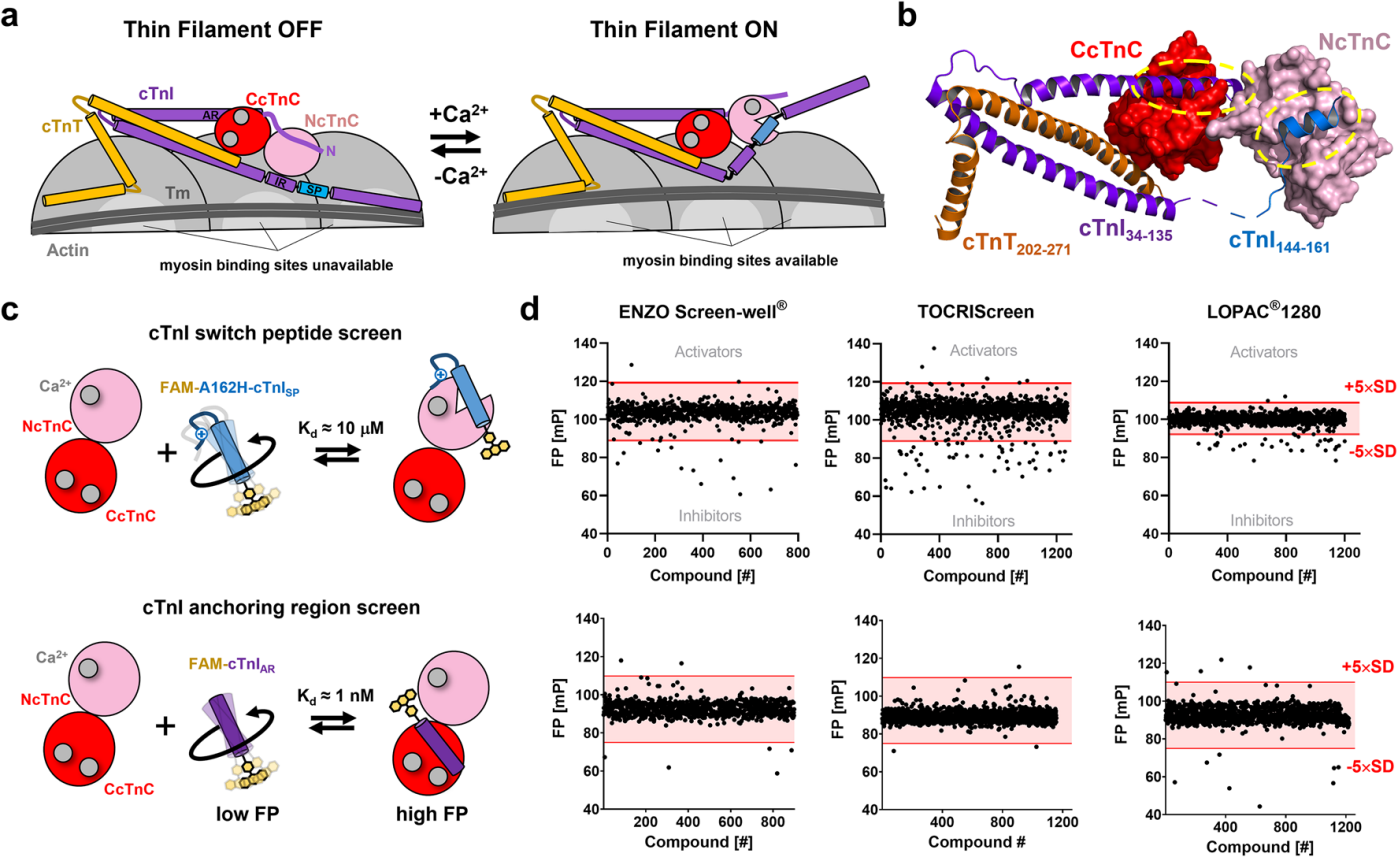
High throughput screens for cardiac troponin modulators. (**a**) Schematic illustration of the calcium-dependent thin filament activation pathway. In the thin filament OFF state (left), myosin binding sites on actin (light gray) are blocked by tropomyosin (Tm; dark gray). Ca^2+^ binding to troponin C (cTnC; red) allows switch peptide (SP; blue) interaction with the regulatory N-lobe of cTnC (NcTnC; pink). Removal of the C-terminal domain of cTnI (CTD) allows Tm to move azimuthally around the thin filament, exposing myosin binding sites on actin. The C-lobe cTnC (CCTnC; red) is constitutively bound to divalent cations and the anchoring region of cTnI (purple). (**b**) Atomic structure of the human cardiac troponin core complex (PDB 1J1D). The N- and C-lobe of cTnC are shown in surface representation in red and pink, respectively. Cardiac troponin I (cTnI) and troponin T (cTnT) are shown in purple and orange, respectively. The switch region of cTnI is shown in blue. Molecular interactions between cTnC and cTnI are highlighted by yellow dashed ovals. (**c**) Cartoon representation of the developed fluorescence polarization (FP)-based HTS that target cTnC-cTnI interactions. Top: FP-based screen targeting the interaction between NcTnC and the switch region of cTnI. Bottom: FP-based screen targeting the interaction between CcTnC and the anchoring region of cTnI. (**d**) HTS of three commercially available compound libraries targeting the interaction between cTnC and switch peptide (top) or the anchoring region of cTnI (bottom). Hits were defined by compounds that change the FP more than five times the standard deviation (5xSD) away from the average value (indicated by red lines).

Cardiac myofilament contraction is initiated by the Ca^2+^-dependent activation of the actin-containing thin filaments. Calcium binding to the troponin complex at the beginning of systole leads to a structural re-arrangement of tropomyosin on the surface of the thin filament that exposes myosin-binding sites on actin (Fig. 1a). Subsequently, myosin heads from the neighboring thick filaments strongly attach to actin and undergo the working stroke coupled to the release of ATP hydrolysis products. The working stroke pulls the thin filaments towards the center of the sarcomere leading to muscle shortening and force generation. Conversely, calcium release from troponin triggers the de-activation of the thin filament, followed by the dissociation of myosin heads from actin and the onset of mechanical relaxation^11^.

The calcium-binding subunit of the trimeric troponin complex, cardiac troponin C (cTnC), can interact with the inhibitory subunit, cardiac troponin I (cTnI), via both its globular N-terminal (NcTnC) and C-terminal (CcTnC) domains, each consisting of two EF-hand motifs^12^ (Fig. 1b). Although, CcTnC is constitutively bound to the anchoring region of cTnI (cTnI_AR_), which anchors cTnC in the troponin complex, Ca^2+^ binding to sites III and IV during the increase in the cytosolic free Ca^2+^ concentration ([Ca^2+^]_i_) at the beginning of systole has been proposed to affect heart muscle function^13^. Moreover, cardiomyopathy-associated mutations in CcTnC have been shown to alter the interaction of cTnC with cTnI_AR_ and modulate myofilament calcium sensitivity^14^, underlining the functional significance of the interaction.

In contrast, NcTnC is found in a closed apo-conformation during low [Ca^2+^]_i_ and Ca^2+^ binding to a single site in NcTnC causes changes in the conformational equilibrium of the domain which allows interaction with switch peptide or switch region of cTnI (cTnI_SP_). Recent advance in cryo-electron microscopy of isolated thin filaments have allowed to characterize the subsequent structural changes at the molecular level. The Ca^2+^-dependent association of NcTnC and cTnI_SP_ has been shown to remove the inhibitory region and C-terminal tail of troponin I of actin and tropomyosin, activating the thin filament^15^.

It follows that the structural OFF/ON transition of the cardiac thin filaments controlled by cTnC-cTnI interactions is a potentially important therapeutic target for the development of compounds with either activating (inotropic) or inhibiting (lusitropic) properties. Such therapeutic interventions would avoid unwanted side effects associated with traditional therapies targeting either the intra-cellular calcium flux or signaling pathways^16^.

However, the development of effective thin filament-directed small molecule effectors has been largely impeded by the lack of screening assays that accurately reproduce cardiac troponin function in vitro. In fact, the majority of currently available small molecule effectors that target cTnC were either identified by homology to other calcium binding proteins of the EF-hand family such as calmodulin^17^, or via phenotypical screenings^18^. Not surprisingly, the majority of identified small molecules either bind other protein targets with much higher affinity (e.g. EMD 57033^19^) or are promiscuous in vivo (e.g. EGCg^20^). Currently only Levosimendan, an NcTnC agonist, is used as part of acute heart failure therapy as an inotropic support and even in this case the molecular targets and mechanisms involved are not fully understood^21,22^.

We have previously reported the development of a high throughput screening (HTS) assay for the calcium-dependent interaction of the cTnI_SP_ with the cTnC^23^. Here, in an analogous approach, we have developed, characterized and validated an HTS assay for the interaction of cTnI_AR_ with the C-lobe of cTnC. The newly developed assay is robust and sensitive, and requires minimal reagents, making it ideal for larger screening campaigns. Using the two developed assay systems targeting either the NcTnC or CcTnC interactions with cTnI, we performed multiple pilot screens of three commercially available compound libraries with more than 3000 compounds in total. Hit compounds were confirmed and validated in secondary screens and orthogonal assays using isolated cardiac myofilaments. Hit compounds-cTnC interactions were characterized using a wide range of biophysical techniques including isothermal titration calorimetry (ITC) and NMR spectroscopy, that allow us to define their interaction modes, binding affinities and interaction sites. Lastly, we tested for the functional effects of compounds in demembranated human myocardium and identified NS5806 as a potential scaffold for the development of new class of calcium sensitizers that stabilize active cardiac troponin. Both the developed HTS assays and identified compounds are suitable platforms for the development of novel cardiac troponin-directed therapeutical interventions for heart failure.

## Results

### Design and validation of high throughput screens that target cardiac troponin

Both the N- and C-terminal lobe of cardiac troponin C have been shown to be the target of small molecule effectors that modulate cardiac myofilament function both in vitro and in vivo^24,25^. We have previously published the design and validation of a high throughput screening (HTS) assay that targets the interaction between the N-terminal lobe of cTnC (NcTnC) and the switch region of cardiac troponin I (cTnI_SP_)^23^. The assay has an excellent dynamic range and signal-to-noise ratio, and the low micromolar affinity of cTnC for cTnI_SP_ allows identification of both interaction inhibitors and activators. In a complementary approach, here we have designed a similar HTS assay for the interaction of the C-terminal lobe of cTnC (CcTnC) with the anchoring region of cTnI (cTnI_AR_) (Fig. 1c). Cardiomyopathy-associated mutations in CcTnC (e.g. Gly159Asp) have been shown to alter its interaction with cTnI_AR_ and thereby modulate myofilament calcium sensitivity^14,26^, underlining the functional significance of this interaction.

To monitor the interaction of cTnC with cTnI_AR_ in-vitro, we have conjugated 6-carboxy-fluoresceine (FAM) to the N-terminus of a peptide corresponding to amino acid residues 39–64 of human cTnI (FAM-cTnI_AR_). Titration of increasing concentrations of cTnC into 1 nmol/L FAM-cTnI_AR_ increased the FP measured from the FAM probe in a concentration dependent manner, indicating protein complex formation (Supplementary Fig. S1). Data points were fitted to a single-site binding model, which gave a K_d_ of about 1 nmol/L. We further confirmed the interaction using Microscale Thermophoresis (MST). Alexa647-labelled cTnC was titrated against increasing concentrations of FAM-cTnI_AR_, which resulted in a binding isotherm with a K_d_ of about 3 nmol/L (Supplementary Fig. S1). The measured K_d_ values are in very good agreement with previously published results using surface plasmon resonance spectroscopy using an unmodified peptide (K_d_ of about 3 nmol/L)^27^, suggesting that FAM-attachment to the designed cTnI_AR_ peptide had no or little effect on its interaction with CcTnC.

We further validated the specificity of the interaction between cTnC and FAM-cTnI_AR_ by titrating increasing concentrations of unlabelled cTnI_AR_ peptide into a mixture of 2 nmol/L FAM-cTnI_AR_ and 5 nmol/L cTnC, and monitored the reaction via changes in FP. The unlabelled peptide decreased the FP in a concentration-dependent manner with an EC_50_ of about 2.6 nmol/L, indicating specific replacement of the FAM-labelled with the unlabelled peptide in the protein complex (Supplementary Fig. S1). Moreover, the interaction of cTnC and FAM-cTnI_AR_ is very stable and was not affected by DMSO concentration of up to 10% (v/v) (Supplementary Fig. S1).

We calculated the Z’-factor of our HTS by adding either DMSO or 50 nmol/L unlabelled cTnI_AR_ peptide to 30 μL assay mixture containing 2 nmol/L FAM-cTnI_AR_ and 5 nmol/L cTnC in 384-well plates^28^ (Supplementary Fig. S1). The calculated Z’-factor and signal-to-noise (S/N) ratio of about 0.8 and 44, respectively, suggest an excellent assay. Moreover, low material requirements (i.e. low nanomolar concentration of reagents) made the developed assay highly cost-efficient and therefore very suitable for HTS applications. However, the high affinity of cTnC for cTnI_AR_ will likely lead to a lower number of hits in primary compound screens and is largely restricted to the identification of inhibitors.

### Pilot screens of commercially available compound libraries and hit validation

Using the developed cTnI_SP_ and cTnI_AR_ HTS assays we performed single concentration screens of three commercially available compound libraries (ENZO Screenwell V2, TOCRIScreen and Sigma LOPAC® 1280) with a total of > 3000 compounds in 384-well format (Fig. 1d). Negative and positive control wells were included in each plate and gave consistent Z’-factors of about 0.8 for each screening plate. Each compound was tested at a concentration of 50 μmol/L and 10 μmol/L for the cTnI_SP_ and cTnI_AR_ screen, respectively. Hits were defined as compounds that changed the average FP value more than five-times the standard deviation (SD) (Fig. 1d, red lines). Moreover, compounds that either increased or decreased the total fluorescence intensity (FI) more than five-times the SD were removed from further analysis, indicating either compound aggregation or unspecific interactions with the fluorophore or protein complex.

**Figure 2:**
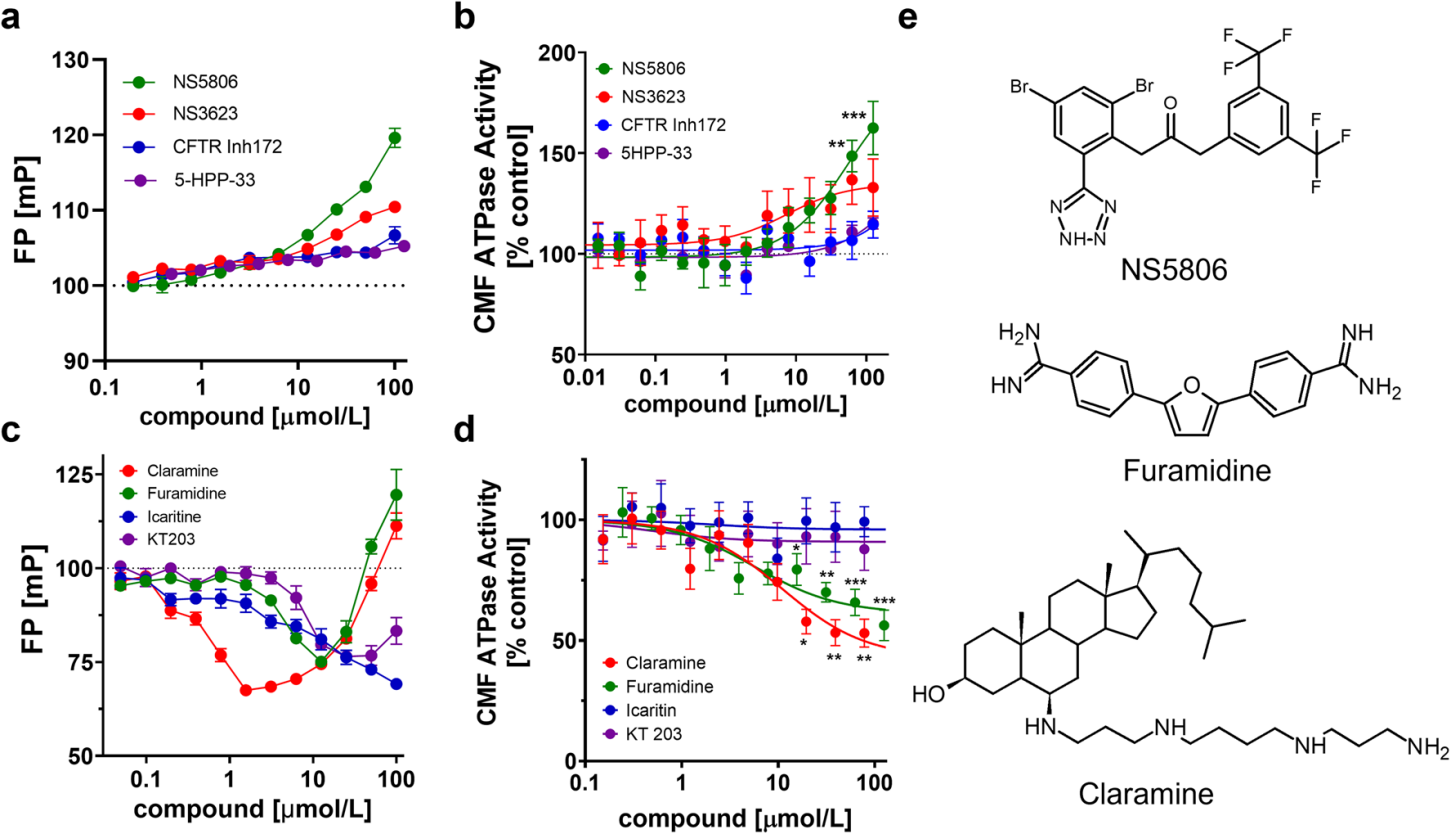
Validation of hit compounds from high throughput screens. (**a**) Dose-dependent effect of selected hit compounds on cTnC-cTnI_SP_ interactions monitored by fluorescence polarization. (**b**) Dose-dependent effect of hit compounds from the cTnC-cTnI_SP_ HTS on the ATPase activity of isolated bovine myofibrils at submaximal calcium activation (~ 30% of maximum activation). (**c**) Dose–response curves for selected hit compounds from the cTnC-cTnI_AR_ HTS. (**d**) Dose-dependent effect of hit compounds from the cTnC-cTnI_AR_ HTS on the ATPase activity of isolated bovine myofibrils at submaximal calcium activation (~ 70% of maximal activation). (**e**) Chemical structures of NS5806, Furamidine and Claramine. Means ± SEM, n = 3–7. Statistical significance of values vs control were assessed with a one-way ANOVA followed by Tukey’s multiple comparison test: *p < 0.05, **p < 0.01, ***p < 0.001.

Although we identified a large number of compounds that decreased the FP values in the cTnC/cTnI_SP_ screen and therefore could act as potential inhibitors of the NcTnC-cTnI switch peptide interaction ^23^ (Supplementary Table S1), we primarily focused on compounds that *increased* the FP from the protein-peptide mixture, indicating increased interaction between cTnC and cTnI_SP_. Such compounds could potentially increase thin filament activation during cardiac systole and could be developed into positive inotropes^18,29^. Of the initial 11 primary hits that significantly increased the FP value, seven could either not be confirmed in secondary assays using higher replicate numbers or were chemically not suitable for further investigations (e.g. calpeptin). The four remaining compounds (NS5806, NS3632, CFTR Inh172 and 5-HPP-33) were further validated using dose–response analysis and an orthogonal assay. All four compounds increased the FP from a mixture of FAM-cTnI_SP_ and cTnC in a concentration dependent manner (Fig. 2a), suggesting an increase in the affinity of cTnC for the FAM-labelled switch peptide, with the strongest increase observed for NS5806 and NS3623 (Fig. 2a). These results were mirrored in an orthogonal assay measuring the ATPase activity of isolated bovine cardiac myofibrils at submaximal calcium activation in the presence of increasing drug concentrations (Fig. 2b). Both NS5806 and NS3623 significantly increased the ATPase activity of cardiac myofibrils in a dose-dependent manner, whereas CFTR Inh172 and 5-HPP-33 showed no or little activation of myofibrillar ATPase in concentrations of up to 100 μmol/L. NS5806 showed the strongest activating effect with an increase in ATPase activity of over 60% with respect to the control at the highest concentration tested. NS5806 was soluble in aqueous buffer solutions in a concentration range of up to 500 μmol/L (Supplementary Fig. S2).

Similarly, we identified 15 primary hits in the cTnI_AR_ screen that decreased the FP from which 11 were excluded based on their chemical properties, exceeding the lower limit of the FP assay or during secondary screens (Fig. 1D) (Supplementary Table S2). The remaining four compounds (Claramine, Furamidine, Icaritine and KT 203) decreased the FP from the cTnC FAM-cTnI_AR_ mixture in a concentration dependent manner, suggesting inhibition of the protein-peptide interaction (Fig. 2c). Surprisingly, we observed a sudden increase in the FP for both Claramine and Furamidine at higher drug concentrations (> 10 μmol/L), potentially indicating assay interference. However, total fluorescence intensity of the assay mixture was not affected by either Claramine of Furamidine at concentrations of up to 100 μmol/L, suggesting that the sudden increase in FP is not caused by compound aggregation (Supplementary Fig. S2). This is further supported by high solubility of both compounds in aqueous solutions (> 25 mmol/L). We tested for the functional effects of the remaining four hit compounds by measuring the ATPase activity of bovine cardiac myofibrils at sub-maximal activation, corresponding to about 70% of the maximal ATPase activity (Fig. 2d). Both Icaritin and KT203 showed no effect on myofibrillar ATPase activity in a concentration range of up to 100 μmol/L and were excluded from further analysis. In contrast, Claramine and Furamidine decreased myofibrilar ATPase activity by about 40–50%.

NS5806, Furamidine and Claramine were chosen for further biophysical and functional characterization. Their chemical structures are shown in Fig. 2e.

### Biophysical characterization of NS5806 binding to cTnC

**Figure 3:**
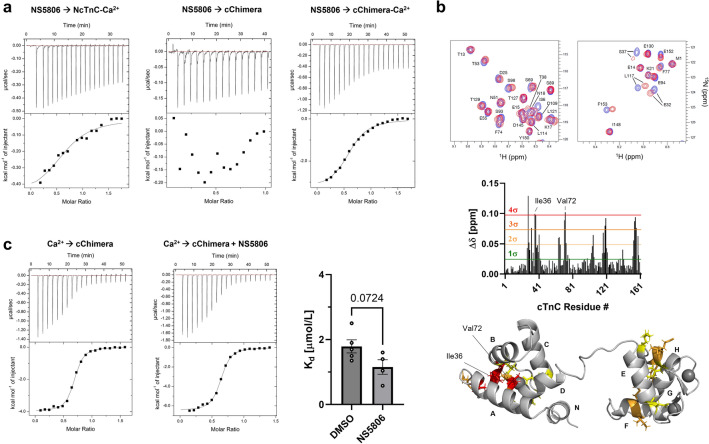
Biophysical characterization of NS5806-cTnC interaction. (**a**) NS5806 binding to Ca^2+^-bound NcTnC (left), Ca^2+^-free cChimera (middle) and Ca^2+^-bound cChimera monitored by isothermal titration calorimetry (ITC). (**b**) Top: Part of 2D-^1^H-^15^N HSQC spectrum of cTnC in the absence (blue) and in the presence (purple) of NS5806 at roughly 1:1 stoichiometry. Middle: Plot of chemical shift perturbations (∆δ) of cTnC plotted against the amino acid sequence after addition of NS5806. Thresholds for different multiples of the standard deviation (SD) are shown in red. Bottom: Chemical shift perturbations mapped onto cTnC structure. Ile36 and Val72 are labelled accordingly. (**c**) ITC binding isotherm for Ca^2+^ titrated into cChimera in the absence (left) and in the presence (right) of NS5806. Means ± SEM, n = 4–5. Statistical significance between control and drug treatment was assessed with an unpaired two-tailed student’s t-test.

Binding of hit compounds to cTnC was confirmed using isothermal titration calorimetry (ITC). Although NS5806 increased cTnI_SP_ binding to cTnC (Fig. 2a), the compound only weakly interacted with the Ca^2+^-saturated N-lobe of cTnC (NcTnC) with an estimated steady-state dissociation constant K_d_ of about 60 μmol/L (Fig. 3a). This is mirrored in the thermodynamic properties with ∆H ≈ − 2 kJ/mol and T∆S ≈ 2.1 kJ/mol. Similarly, NS5806 only weakly bound to a Ca^2+^-free cChimera construct consisting of NcTnC C-terminally conjugated to the switch region of cTnI^30^, which, however, could not be reliably quantified using ITC. In contrast, NS5806 showed very clear saturable binding to the Ca^2+^-saturated cChimera with a K_d_ of 7.6 ± 0.3 μmol/L and stoichiometry of about 1:1 (means ± SEM, n = 4). Moreover, the interaction has both favorable enthalpic (∆H = −11.4 ± 0.8 kJ/mol) and entropic contributions (T∆S = 17.6 ± 0.9 kJ/mol), suggesting that the NS5806-cChimera complex is stabilized by both hydrophilic and hydrophobic interactions. These results suggests that NS5806 occupies a binding pocket formed by both the Ca^2+^-saturated NcTnC and cTnI_SP_. In good agreement, NS5806 shows clear saturable binding to Ca^2+^-saturated NcTnC in the presence of the untethered cTnI switch peptide with a K_d_ of about 19 μmol/L (Supplementary Fig. S3).

We mapped the NS5806 binding site on Ca^2+^-saturated cTnC using two-dimensional ^1^H-^15^N-HSQC NMR spectroscopy (Fig. 3b). NS5806 titration into cTnC gave modest chemical shift perturbations (∆δ, CSPs) between 0.1 and 0.15 ppm with the binding occurring in the fast-exchange regime suggesting fast binding/unbinding kinetics. For NS5806 the largest CSPs are clustered tightly around the hydrophobic residues Ile36 and Val72 of the antiparallel β-sheet and the adjacent loops of the N-lobe, next to the calcium binding site II, suggesting that NS5806 binds deeply into the hydrophobic groove of Ca^2+^-NcTnC. In contrast, lower CSPs are seen in the C-lobe in all four helices. No significant CSPs for the hydrophobic residues Ile112 and Ile148 of the central β-sheet indicates that NS5806 is not buried deep in the hydrophobic center of CcTnC and likely only weakly interacts with its the surface. A very similar behavior has been reported for the inhibitor W-7, which interacts with both the N-lobe and C-lobe of isolated cTnC, but exclusively binds to NcTnC in the presence of cTnI, suggesting unspecific interaction with the hydrophobic groove of CcTnC^31,32^.

We further tested for the effects of NS5806 on the Ca^2+^ binding affinity of cChimera (Fig. 3c). In the absence of NS5806, cChimera binds Ca^2+^ with a K_d_ of 1.8 ± 0.2 μmol/L (mean ± SEM, n = 5), which is significantly lower than the K_d_ previously reported for NcTnC alone using ITC (K_d_ of ~ 15 mmol/L) but higher than for cChimera using NMR spectroscopy^30,33^. In the presence of NS5806 the Ca^2+^-affinity of cChimera is slightly increased with a K_d_ of 1.2 ± 0.2 μmol/L, which however, did not reach statistical significance (*p* = 0.07 for an unpaired, two-tailed student’s t-test).

### Biophysical characterization of Furamidine binding to cTnC

**Figure 4:**
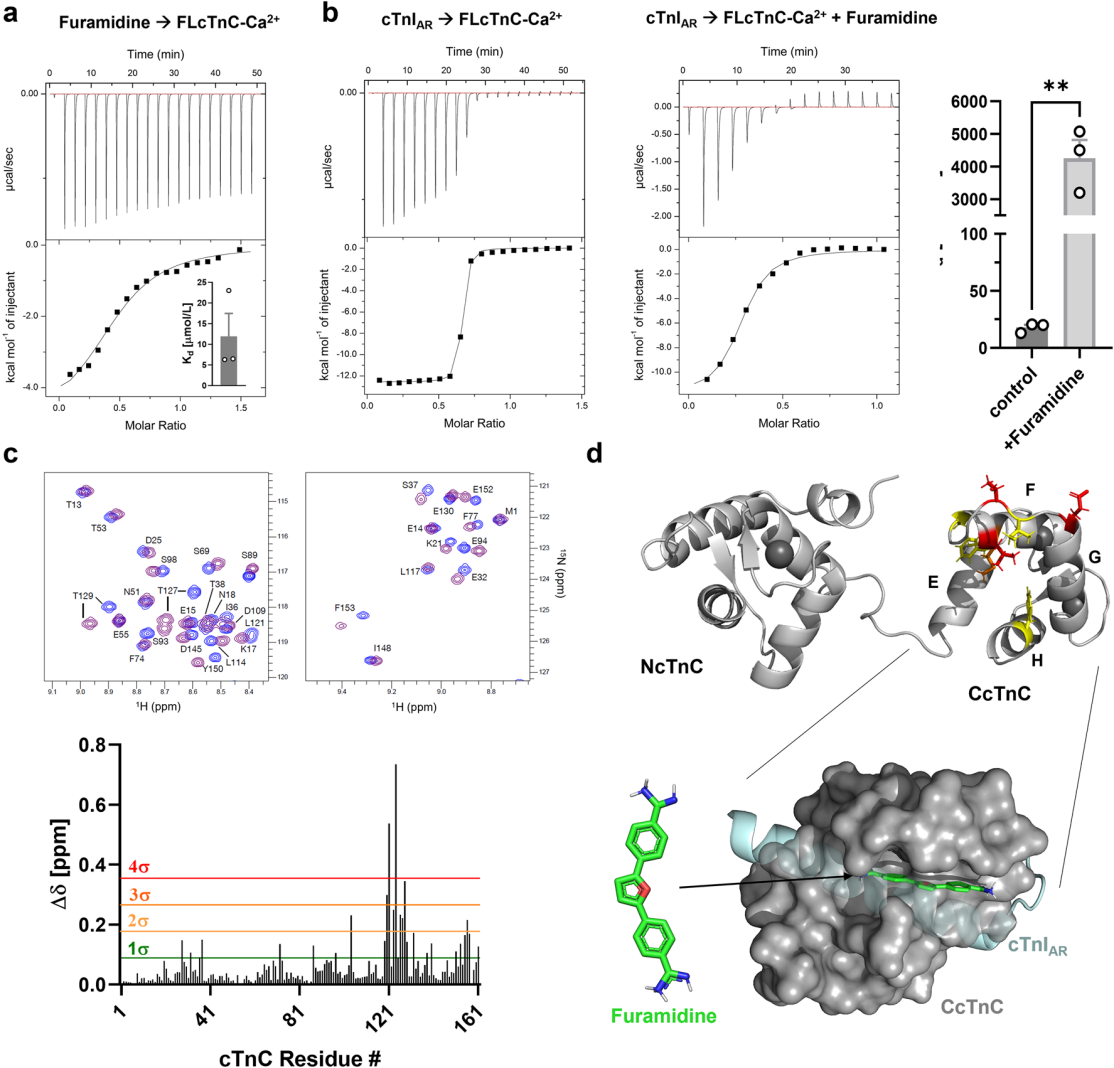
Biophysical characterization of Furamidine-cTnC interaction. (**a**) ITC for titration of Furamidine into cTnC. Insert: Measured steady-state dissociation constants. (**b**) ITC titration of unmodified cTnI_AR_ into cTnC in the absence (left) and in the presence of Furamidine (middle). Measured steady-state dissociation constants are shown on the right. (**c**) Top: Part of 2D-^1^H-^15^N HSQC spectrum of cTnC in the absence (blue) and in the presence (purple) of Furamidine. Bottom: Plot of chemical shift perturbations (∆δ) of cTnC after addition of excess Furamidine. (**d**) Top: Chemical shift perturbations mapped onto cTnC structure with the same colour coding as shown in (**c**). Bottom: Docking pose for Furamidine (green) binding to CcTnC (grey, surface representation) using flexible docking in AutoDock Vina. The position of cTnI_AR_ (cyan) in the cardiac troponin complex (PDB 1J1D) was superimposed onto the structure. Means ± SEM, n=3. Statistical significance between control and drug treatment was assessed with an unpaired two-tailed student’s t-test: **p<0.01.

The ITC isotherm for Furamidine binding to full length cTnC is shown in Fig. 4a. Furamidine binds Ca^2+^-saturated cTnC with a roughly 1:1 stoichiometry and a K_d_ of 11.9 ± 5.5 μmol/L (means ± SEM, n = 3). The interaction has both a favorable enthalpic (∆H = −19.4 ± 3.17 kJ/mol) and entropic contributions (T∆S = 7.7 ± 3 kJ/mol).

Furamidine was discovered in the primary screen as a potential inhibitor of the cTnC-cTnI_AR_ interaction and we quantified its effect on the protein-cTnI_AR_ peptide interaction using ITC. Titration of an unlabelled cTnI_AR_ peptide into cTnC gave a well-defined and saturable binding isotherm with a K_d_ of 17.9 ± 2.6 nmol/L (means ± SEM, n = 3) (Fig. 4b), in agreement with the binding affinities estimated by both FP and MST described above (K_d_ of 1–3 nmol/L). The small differences in the measured affinities of cTnC for the cTnI_AR_ between the different assays can likely be attributed to the different buffer conditions used in each binding assay. Nonetheless, in the presence of excess Furamidine the binding affinity of cTnC for cTnI_AR_ is reduced by several orders of magnitude as indicated by a K_d_ of 4.2 ± 0.5 μmol/L (Fig. 4b, left), suggesting that Furamidine either reduces the affinity of cTnC for the anchoring region of cTnI or directly competes for the same interaction site.

We mapped the Furamidine binding site on cTnC using two-dimensional ^1^H-^15^N-HSQC NMR spectroscopy. A small section of the HSQC spectrum of free cTnC (blue) superimposed with the spectrum at about 1:1 molar ratio of added Furamidine (purple) is shown in Fig. 4c. Furamidine binding occurs in the fast-exchange regime, suggesting fast binding/unbinding kinetics of the drug to the protein. Quantitative analysis of the chemical shift perturbations (CSPs) shows a cluster of highly significant values in only the C-lobe of cTnC around residues 120–130 (Fig. 4c, bottom), suggesting that Furamidine does not interact with NcTnC. Mapping of the CSPs to the Ca^2+^-bound structure of cTnC (PDB 1AJ4) shows a defined interaction surface in the C-lobe distal to the calcium binding sites III and IV (Fig. 4d, top). No significant CSPs for the hydrophobic residues Ile112 and Ile148 of the central β-sheet indicates that Furamidine is not buried deep in the hydrophobic pocket of CcTnC. Using the CSPs to define the interaction surface, we performed flexible docking in AutoDock Vina to determine Furamidine’s potential binding modes (Fig. 4d, bottom)^34^. The in-silico results suggest that Furamidine sits in the hydrophobic groove of the cTnC C-lobe, which is normally occupied by the anchoring region of cTnI in the troponin complex (Fig. 4d, cyan helix). Taken together these results suggest that Furamidine might directly compete with cTnI_AR_ for the same binding site in CcTnC.

### Biophysical characterization of Claramine binding to cTnC

**Figure 5:**
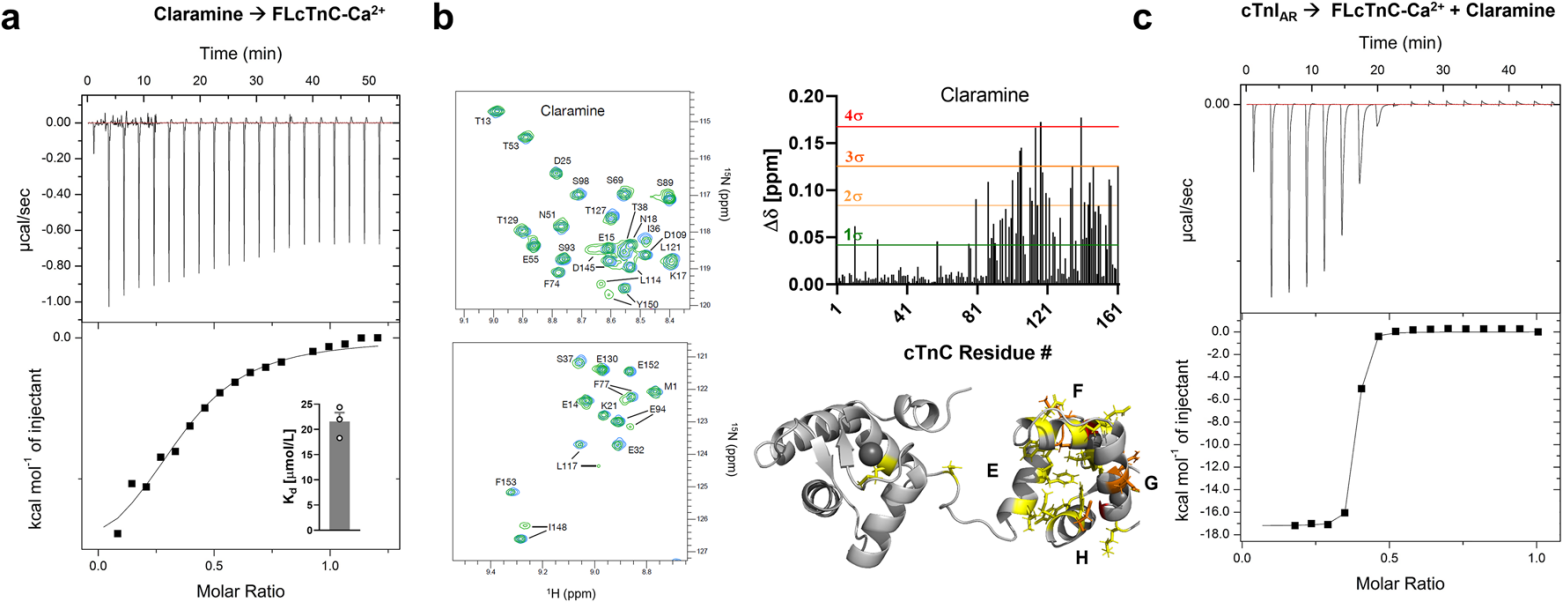
Biophysical characterization of Claramine-cTnC interaction. (**a**) ITC for titration of Claramine into cTnC. Insert: Measured steady-state dissociation constants. (**b**) Left: Part of 2D-^1^H-^15^N HSQC spectrum of cTnC in the absence (blue) and in the presence (purple) of Claramine. Right: Plot of chemical shift perturbations (∆δ) of cTnC after addition of excess Furamidine. Chemical shift perturbations mapped onto cTnC structure. (**c**) ITC titration of unmodified cTnI_AR_ into cTnC in the presence of Claramine (middle). Please note that Claramine had no effect on the cTnC-cTnI_AR_ interaction.

Claramine binds to isolated cTnC with K_d_ of 21.6 ± 1.8 μmol/L (means ± SEM, n = 3) (Fig. 5a), which is about two-times weaker compared to Furamidine. The weaker binding is mirrored in the thermodynamic parameters (∆H = −4.6 ± 1.3 kJ/mol and T∆S = 21.8 ± 1.3 kJ/mol) and suggests a mostly entropy-driven interaction likely mediated via hydrophobic interactions of its steroid group with the hydrophobic pocket of CcTnC.

Titration of Claramine into cTnC was monitored via ^1^H-^15^N-HSQC NMR spectroscopy to determine its binding site (Fig. 5b). Claramine only produced modest CSPs in cTnC, although in the slow exchange regime, which were mostly localized to its C-terminal domain but over a broad interaction surface surrounding the hydrophobic pocket. Similar to Furamidine, small or no CSPs for residues Ile112 and Ile148 suggest that Claramine is not buried deep in the hydrophobic cleft of CcTnC and likely resides near its opening. Interestingly, the largest CSPs were observed for residues Asp113 and Glu116, which are part of CcTnC Ca^2+^-binding site III. In good agreement with the relatively weak and potentially unspecific interaction, Claramine had no or little effect on the interaction between the cTnC and the anchoring region of cTnI as measured by ITC (Fig. 5c). The estimated steady-state dissociation constants K_d_ in the presence of Claramine was about 40 nmol/L, which is only marginally higher compared to the control in the absence of the drug.

### Functional effects of hit compounds on the calcium sensitivity of human heart tissue

**Figure 6:**
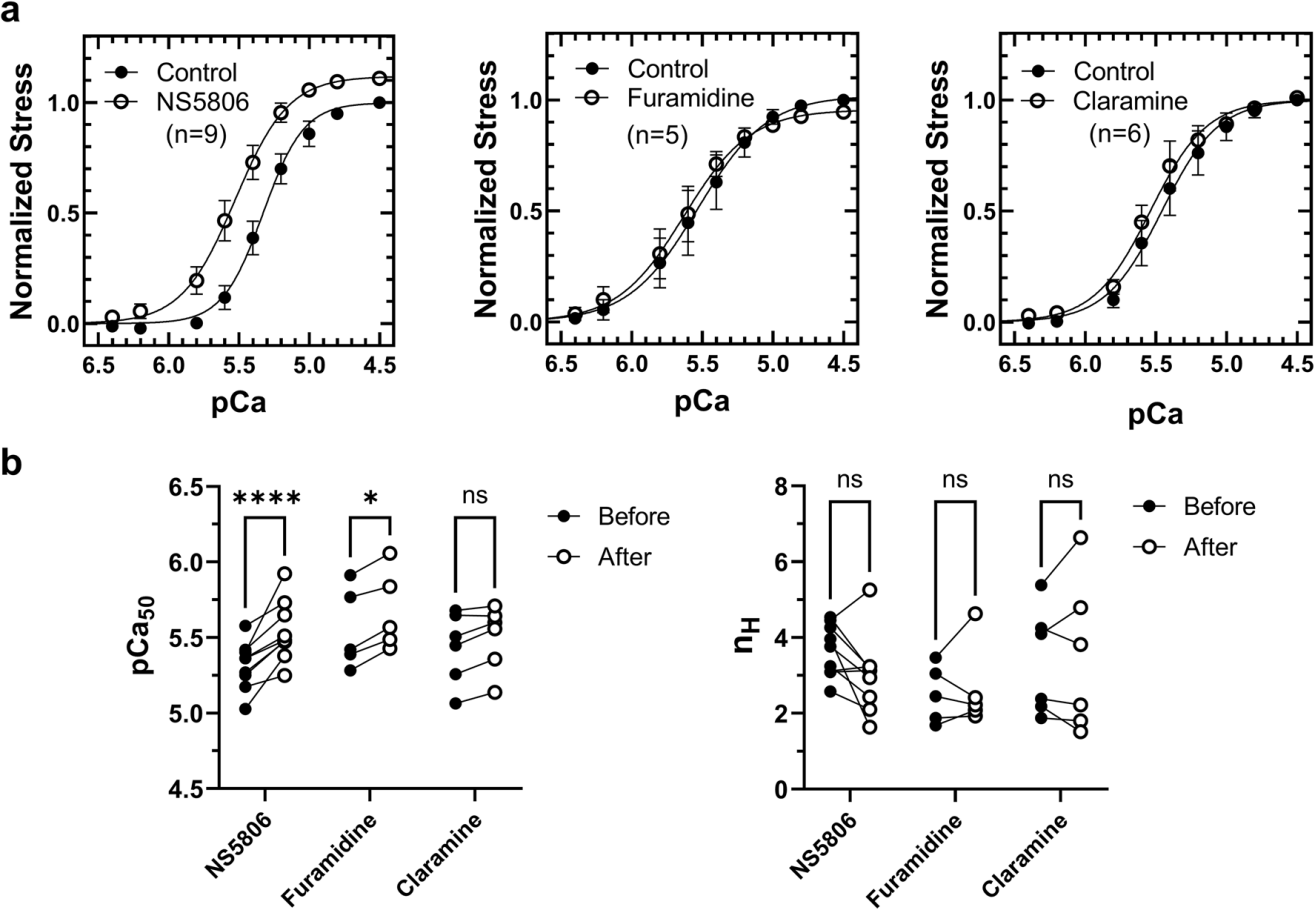
Functional effects of hit compounds in demembranated human myocardium. (**a**) Force-pCa relations of demembranated human myocardium in the absence and in the presence of 100 μmol/L NS5806 (left), Furamidine (middle) and Claramine (right). (**b**) Effects of hit compounds on calcium sensitivity (pCa_50_) and cooperativity (n_H_) of force development of demembranated human myocardium. Means ± SEM, n = 5–9. Statistical significance of differences between before and after drug treatment were analyzed by a two-way ANOVA followed by Sidak’s multiple comparison test: *p < 0.05, ****p < 0.0001, ns—not significant.

We tested for the functional effects of hit compounds by measuring the calcium sensitivity of force development of chemically demembranated human donor ventricular tissue at 2 μm sarcomere length in the absence and in the presence of 100 μmol/L compounds (Fig. 6). Force-pCa (pCa =  −log_10_[Ca^2+^]) relations for each preparation was measured in the absence and in the presence of each compound, so that each muscle preparation served as its own control. Time-matched control experiments showed no significant differences in the calcium sensitivity as indicated by pCa_50_ (pCa_50_ = −log_10_[Ca^2+^] for half maximal activation), steepness of the force-pCa relation (n_H_) and maximal active isometric force after the first and second force-pCa titration in the absence of drugs (Supplementary Fig. S4), suggesting very little preparation rundown. Measured pCa_50_ and n_H_ values for demembranated human donor samples are in very good agreement with previously published results^35^.

NS5806 strongly increased the calcium sensitivity of force development of demembranated human muscle strips as indicated by an increase in pCa_50_ by about 0.2 pCa. Similarly, there was a trend towards a lower steepness of the force-pCa relation as indicated by the Hill coefficients (n_H_), which however, did not reach statistical significance (Fig. 6a and b). Moreover, NS5806 increased the maximal active isometric force at full calcium activation (pCa 4.5) by about 10%. The net effect is a large increase in the active force development of human cardiac muscle at calcium concentrations close to the value measured in intact muscle cells during systole (pCa 5.8–6). The functional effects of NS5806 are in very good agreement with the idea that the compound stabilizes the interaction between cTnC, Ca^2+^ and the switch region of cTnI in-situ and therefore acts as a calcium sensitizer of the contractile myofilaments.

The increase in calcium sensitivity and maximal isometric force by NS5806 was also preserved at longer sarcomere length (Supplementary Fig. S5), suggesting that the compound did not affect the length-dependent activation response of human donor myocardium. Moreover, we have tested for the functional effects of NS5806 in non-ischemic heart failure tissue with very low cardiac troponin I and myosin binding protein-C phosphorylation levels^36^. Intriguingly, although NS5806 did increase the calcium sensitivity of the failing human tissue, it had no effect its maximal isometric force.

100 μmol/L Furamadine treatment slightly increased the calcium sensitivity of demembranated human cardiac muscle strips but had no effect on the steepness of the force-pCa relation or maximal isometric force development (Fig. 6a and b). It is likely that the high binding affinity and local concentrations of CcTnC and cTnI_AR_ in the myofilaments cannot be overcome by small molecule effectors that bind CcTnC with only micromolar affinity in the concentration range tested (≤ 100 μmol/L). In contrast, Claramine had no or little effect on the force-pCa relation of demembranated human cardiac muscle cells.

## Discussion

The myofilaments have become an attractive therapeutic target for the treatment of both cardiomyopathies and heart failure^25,37^, potentially increasing efficacy and avoiding unwanted side effects associated with current therapies. Recently, the cardiac myosin-specific inhibitor Mavacamten (now known as Camzyos™) was approved by the FDA for the treatment of obstructive HCM^38^.

In this study, we developed novel HTS assays that directly target the regulatory interactions between the cTnC- and cTnI-subunits of the cardiac troponin complex. The assays are robust and sensitive, and identified biological active compounds even from small compound libraries. Moreover, hit rates of about 0.13% after secondary screens and about 0.07% after orthogonal assays suggests manageable number of compounds even with very large libraries (> 100,000 compounds).

We identified NS5806 as a novel calcium sensitizer of the cardiac myofilaments, which binds to and stabilizes the trimeric complex of NcTnC, Ca^2+^ and the switch region of cTnI. Surprisingly, NS5806 had only little effect on the Ca^2+^-affinity of the isolated N-lobe of cTnC fused to the switch peptide of cTnI (i.e. cChimera) (Fig. 3c), suggesting that the increased calcium sensitivity of demembranated human cardiac muscle fibres in the presence of NS5806 is likely not due to an increase in Ca^2+^-binding affinity per se, as observed for other calcium sensitizers targeting cardiac troponin ^39^, but increased affinity of cTnI_SP_ for Ca^2+^-bound NcTnC (Fig. 2a). However, the current results cannot exclude that NS5806 increases the calcium affinity of the cardiac troponin complex bound to the thin filaments in situ.

In contrast to skeletal TnC, NcTnC remains mostly in its closed conformation in the Ca^2+^-bound state and only adopts the fully open conformation in the presence of the cTnI switch peptide^40^. Similarly, the cChimera has recently been shown to adopt its closed conformation in the absence of Ca^2+^^41^. Taken together with the weak binding affinities of NS5806 for both isolated NcTnC and calcium-free cChimera (Fig. 3a), these results strongly suggests that NS5806 occupies a binding pocket created by the interface between the open conformation of NcTnC and cTnI switch peptide.

NS5806 has been shown to act as an activator of voltage-gated potassium channel K_v_4.3 by binding to potassium channel-interacting proteins (KChIPs) with low micromolar affinity and increase their affinity for the channel^42^. KChIPs are calcium-binding proteins containing several EF-hand motifs in their C-terminal domain and NS5806 has been shown to bind close to the hydrophobic pocket near EF-hand 4 of KChIP3 in the Ca^2+^-bound state^42^. KChIP1 bound to a fragment of Kv4.3 (PDB 2I2R) shows a high degree of structural similarity with the Ca^2+^-saturated NcTnC bound to the switch peptide of cTnI (PDB 1MXL) with an RMSD < 1.7 Å (Supplementary Fig. S6). This suggests that NS5806 might occupy a similar hydrophobic pocket created by the NcTnC-cTnI_SP_ interface and stabilize this interaction. The large enthalpic contribution of NS5806 binding to cChimera, however, excludes an exclusively hydrophobic interaction, although a large exothermic structural re-arrangement upon NS5806 binding cannot be excluded. Of note, although NS5806 significantly increased the affinity of KCHiP3 for K_v_4.3, it had only a marginal effect on its Ca^2+^-affinity, which is similar to the ITC data for Ca^2+^-titration into cChimera in the presence of NS5806 and suggests a potentially similar mechanism of action.

A compound with similar binding properties, RPI-194, was recently identified using an NMR-based structural screening approach^43^. RPI-194 weakly binds the isolated Ca^2+^-saturated N-lobe of cTnC (K_d_ > 300 μmol/L) but shows low micromolar affinity to a Ca^2+^-saturated cChimera. However, the functional effects of NS5806 on demembranted cardiac muscle fibres (i.e. increase in maximal force production) are different to those reported for the troponin activator RPI-194^43^, suggesting that both molecules stabilize activated troponin via distinct mechanisms. In contrast, NS5806 has similar functional properties to TA1^18^, a cardiac troponin agonist currently undergoing Phase I clinical trials, albeit with lower potency. Although the origin of the increased maximal isometric force production by NS5806 (and TA1) is currently unknown, it is likely that even at full Ca^2+^-activation (pCa 4.5) not all thin filament regulatory units in demembranated cardiac muscle fibres are in the fully ON state and that addition of the troponin modulators completely activate the thin filaments. Alternatively, the increased force production might be related to off-target effects of these compounds.

We performed a structural similarity analysis of NS5806 to known cardiac troponin C calcium sensitizer using multi-dimensional scaling in ChemMine Tools^44^ (Supplementary Fig. S7). The results show that NS5806 did not cluster with any of the known troponin activators, suggesting that the compound is a potential novel chemical scaffold for the development of heart failure therapeutics.

Although Furamidine did not have a strong functional effect on cardiac myofilament function, it is likely a useful scaffold for the development of future lead compounds that target the C-lobe of cTnC. The only other known compound that has been shown to specifically interact with the C-lobe of cTnC is the calcium sensitizer EMD 57033, which binds CcTnC with micromolar affinity^45,46^. However, in contrast to Furamidine, EMD 57033 had no significant effect on the CcTnC and cTnI_AR_ interaction in vitro as measured by NMR spectroscopy, in stark contrast to Furamidine which significantly lowered the affinity by several orders of magnitude. EMD 57033 sits deep in the hydrophobic pocket of CcTnC as evident by contacts between Ile112 and Ile148 with its thiadiazone group and stabilizes a more open conformation of the domain by contacts with residues Leu117 and Leu121 on the F-helix that pull it away from helix E to helix G. These structural rearrangements are similar to the effects of cTnI_AR_ binding to CcTnC^47^, indicating that these changes are a pre-requisite for the interaction of the two proteins. In contrast, similar contacts are largely missing for Furamidine (Fig. 4c), suggesting that it does not stabilize the open conformation of CcTnC. Furamidine binding to CcTnC might, in fact, stabilize its closed conformation, which might be the molecular basis of its inhibitory effect on the interaction between CcTnC and the cTnI anchoring region.

Taken together, our results suggest that sarcomere-directed targeted screening strategies are suitable to identify biologically active compounds that can be developed into new heart failure therapeutics.

## Methods

### Production of proteins and peptides

Full length human cardiac troponin C (cTnC) and its N-terminal domain (NcTnC), and human cChimera construct containing NcTnC C-terminally conjugated to the switch region of cardiac troponin I (cTnI) were expressed from a modified pET6a vector in BL21 (DE3)-RIPL cells (Agilent Technologies Inc.) fused to a hexa-histidine tag and TEV protease site. Proteins were purified on HisTrapFF columns (GE Heatlhcare), and histidine-tag was removed by treating proteins overnight with 1:100 stoichiometry of hexahistidine-tagged TEV protease. TEV protease and histidine-tag were removed by applying the mixture onto a 1 ml HisTrapFF column, and the flow-through containing the purified protein was collected. Protein purity was estimated to be > 95% by SDS-PAGE and electron spray ionization mass spectrometry (ESI–MS). The N-terminally 6-carboxy-fluoresceine (FAM) labelled A162H-mutated switch region peptide of human cardiac troponin I (sequence: TLRRVRISADAMMQALLGARHKESLDLR) and the N-terminally FAM-labelled anchoring region peptide of human cardiac troponin I (FAM-cTnI_AR_; sequence: SKISASRKLQLKTLLLQIAKQELERE) were purchased from Fisher Scientific (> 99% purity, TFA removed).

Protein expression with ^15^N isotope labelling was performed as described previously^48^. The protein was purified on a 5 mL HisTrap FF6 column as per the manufacturers protocol. The his-tag was subsequently cleaved using TEV protease and removed along with other contaminating proteins by a second application on the 5 mL HisTrap FF6 column. Purity of the protein was judged by SDS-PAGE to be > 95%. The purified protein was buffer exchanged using a PD10 desalting column and concentrated using a Vivaspin 20 concentrator with 5 kDa cutoff. All measurements were carried out in NMR buffer (composition in mmol/L: 20 HEPES pH 7.2, 100 NaCl, 5 CaCl_2_, 2 DTT, 0.02% (w/v) NaN_3_) with 10% (v/v) D_2_O in a total volume of 350 μL using a Shigemi tube.

### High throughput screening and fluorescence polarization assays

The high throughput screen protocol using the FAM-labelled cTnI_SP_ has been previously described^23^. Libraries were reformatted from 96-well plates to Greiner 384-well black plates using a GILSON PipetMax. The plates were sealed with adhesive foil and stored at − 20 °C until further use. 30 μL of master mix containing 2 nmol/L FAM-cTnI_AR_ and 5 nmol/L cTnC in assay buffer (20 mmol/L Tris–HCl pH 7, 100 mmol/L NaCl, 1 mmol/L CaCl_2_, 1 mmol/L DTT) were added to each well of a black Greiner 384-well plate, and either DMSO or 50 nmol/L unlabelled cTnI_AR_ peptide were added to two columns per plate as negative and positive controls, respectively. A total of 40 nL of 10 mmol/L compound stocks were transferred to each well using a Mosquito liquid handler system (final DMSO concentration of 0.1% (v/v)). The plates were incubated in the dark for 30 min at 25 °C, and the fluorescence polarization from each well was measured using a ClarioStar Plate reader with appropriate excitation and emission filter settings. Dose–response curves were constructed by serial dilution of compounds in DMSO and adding a fixed volume to a mixture of peptide and cTnC.

Fluorescence polarization dose–response and binding experiments were performed in Greiner 384-well black plates in assay buffer (20 mmol/L Tris–HCl pH 7, 100 mmol/L NaCl, 1 mmol/L CaCl_2_, 1 mmol/L DTT) at 25 °C.

### Myofibrilar ATPase activity measurements

Bovine cardiac myofibrils (CMF) were freshly prepared from bovine ventricle as previously described^49^. The NADH-coupled ATPase assay was adapted to test the effects of drugs on bovine cardiac myofibrils^50^. Briefly, buffers with varying free [Ca^2+^] were prepared by adding the appropriate concentrations of EGTA and CaCl_2_ calculated using the MAXCHELATOR software. ATPase assays for hits identified in the cTnI switch peptide and anchoring region screens were performed at pCa 6 and pCa 5.8, respectively. A substrate mix (composition: 220 µmol/L NADH, 2 mmol/L phospho-enolpyruvate and 2 mmol/L ATP) and an enzyme mix (composition: 0.5 mg ml^−1^ myofibrils, 40 U ml^−1^ Lactate Dehydrogenase and 200 U ml^−1^ Pyruvate Kinase) were prepared for a final reaction volume of 40 μL. 20 μL Enzyme mix was added to individual wells using a 12-well multichannel pipette. Appropriate concentrations of drugs were added to each well and the plate was shaken at 1000 rpm for 5 min on a plate shaker and further incubated at room temperature for 10 min. Substrate mix was dispensed into each well using the Gilson PipetMax. The plate was briefly spun down to ensure removal of air bubbles. The reaction was followed by recording the fluorescence intensity of each well with excitation at 380 nm and emission at 470 nm, for 10 min scanning the plate every 30 s. The temperature was kept constant at 25 °C. ATPase rates were extracted by linear regression to datapoints in GraphPad Prism.

### Isothermal titration calorimetry

Thermodynamic parameters for the appropriate drugs binding to NcTnC, cChimera and cTnC were determined in ITC buffer (composition in mmol/L: 20 MOPS, 100 KCl, 1 MgCl_2_, pH 7) without or with 1 mmol/L CaCl_2_ using a MicroCal ITC200 titration calorimeter (Malvern Panalytical). The protein stock solutions were dialysed against ITC buffer overnight. The sample cell and injection syringe were extensively cleaned with decalcified water and then extensively washed with ITC buffer. The reaction cell was loaded with 180 µmol/L NcTnC, 200 µmol/L cTnC or 150 μmol/L cChimera in the appropriate buffer. Drug or peptides solutions were manually loaded into the syringe and titrated into the reaction cell containing the protein solutions with 3 min intervals over 20–40 injections. The first injection was ignored and corrections for the heat of dilution was made by subtracting the last injection from all prevous injection points. The temperature was kept constant at 25ºC, and stirring speed was at 1000 rpm. ITC data were analyzed using Origin 7 ITC data analysis software (OriginLab Corp., Northampton, MA).

### Microscale thermophoresis

MST experiments were performed on a Monolith NT.115 instrument (NanoTemper) in interaction buffer containing 20 mmol/L MOPS, pH 7, 1 mmol/L CaCl_2_, 50 mmol/L KCl, 1 mmol/L DTT and 0.05% (v/v) Tween-20. Human cTnC was labelled with Alexa 647-NHS (Molecular Probes, Inc; ThermoFisher Scientific) according to the manufacturer’s instructions, and dye incorporation (efficiency of > 80%) was confirmed by HPLC and ESI–MS. Labelled cTnC was gel-filtered into the interaction buffer. Titration experiments were performed with a fixed concentration of 100 nmol/L of Alexa647-labelled cTnC in premium capillaries at a constant temperature of 25 °C.

### NMR spectroscopy

All NMR experiments were carried out on a Bruker Avance III 800 MHz spectrometer at T = 298 K. Standard ^1^H-^15^N HSQC experiments with flip back pulse and watergate were used as provided by the manufacturer. Spectra were recorded with spectral widths of 15.61 and 27 ppm for ^1^H and ^15^N, respectively using 2048 and 250 points giving a FID resolution of 12.2 and 17.5 Hz. Data was collected with 32 scans preceded by 8 dummy scans. Spectra were processed using Bruker Topspin 4.1.4 to a matrix of 4096 × 2048 complex points. FIDs were apodized with Sine2 window functions shifted by 3 and 2.5. Water suppression was enhanced using Qfil followed by a polynomial baseline correction. Ligands were dissolved in DMSO at a stock concentration of 50 mM. Appropriate quantities of stock were added to the NMR samples to reach the desired concentration.

Processed spectra were analysed in CCPNMR 2.4.2 and 2.5^51^. Backbone amide assignments for full length human cardiac TnC were obtained from combining BMRB entries 4994 and 25,797 supplemented by a ^15^N 3D-NOESY-HSQC experiment to resolve ambiguities. Out of 161 amino acids in the construct only three (K6, A7, M85) could not be assigned in this way. For interaction mapping reference HSQC experiments were recorded with protein concentrations of 380 μmol/L (NS5806) and 200 μmol/L (Furamidine, Claramine). Ligand binding experiments were recorded over a range of ligand concentrations from 0.5:1 to 3:1. It was found that the quality of the spectra was best around 1:1 (ligand:protein) and these spectra were analysed in full to determine the chemical shift perturbations (CSP) for each assigned amide group. Peak assignment in the spectra with added ligands was done by following the peaks moving as more ligand was added in cases of fast exchange (Furamidine, NS5806). In the case of slow exchange an exchange experiment was recorded to link the new species appearing at increasing ligand concentration in addition to the peak of the free protein. Where the exchange experiment did not produce useful data the minimal CSP approach was used.

CSPs were extracted and plotted against the sequence using Apple Numbers. Simple standard deviation was calculated for each of the three plots and used as significance thresholds. Levels from 1*sigma to 4*sigma were created and used as cutoffs for colouring of 3D models of TnC alone (PDB code: 1AJ4) or in the Tn complex (PDB code: 1J1E). 3D protein structure images were generated in Pymol.

### Preparation of human cardiac muscle strips and force-Ca^2+^ titrations

Human samples were obtained after informed consent and with approval of the institutional review board of the University of Kentucky (08-0338-F2L; approval date: January 18, 2017) and the King’s College London Research Ethics Panel (LRM-20/21-22235; approval date: 28th September 2021). This investigation conformed with the principles of the Declaration of Helsinki (1997). Mechanical experiments and force-calcium titrations were performed as previously described^35^. Briefly, each demembranated cardiac muscle strip underwent a control force-pCa titration in the absence of compounds with the sarcomere length adjusted to about 2 µm by laser diffraction. Drugs were freshly diluted in DMSO and added to physiological buffer solutions. The final DMSO concentration was held constant at 0.2% (v/v). Subsequently, demembranated human heart muscle strips were equilibrated in relaxing solution (pCa 7) containing the appropriate concentrations of drugs for 25 min at 22 °C and the force-pCa titration repeated. Thus, each cardiac muscle strip served as its own negative control and force data were normalized to the maximal force of each preparation in the absence of compounds.

## Supplementary Information

Supplementary Information.

## Data Availability

All data are contained within the manuscript and supplementary information. Source files can be obtained from the corresponding author upon reasonable request.
