# Supplementary material for: Discovery of novel cardiac troponin activators using fluorescence polarization-based high throughput screening assays
Source: Sci Rep. 2023 Mar 30;13:5216. doi: 10.1038/s41598-023-32476-w (PMC10063609; doi:10.1038/s41598-023-32476-w)
Supplement: Supplementary file 1 — Supplementary Information. [file 41598_2023_32476_MOESM1_ESM.pdf]

Supplementary Information

Discovery of Novel Cardiac Troponin Activators using Fluorescence Polarization-based High Throughput Screening Assays

Priyanka Parijat[1], Saraswathi Ponnam[1], Seetharamaiah Attili[1], Kenneth S. Campbell[2], Mohammed El-Mezgueldi[1], Mark Pfuhl[1] and Thomas Kampourakis[1,*]

[1]Randall Centre for Cell and Molecular Biophysics; and British Heart Foundation Centre of Research Excellence, King's College London, London, SE1 1UL, United Kingdom

[2]Department of Physiology, and Division of Cardiovascular Medicine, University of Kentucky, Lexington, Kentucky

Supplementary Information Figures and Figure Legends

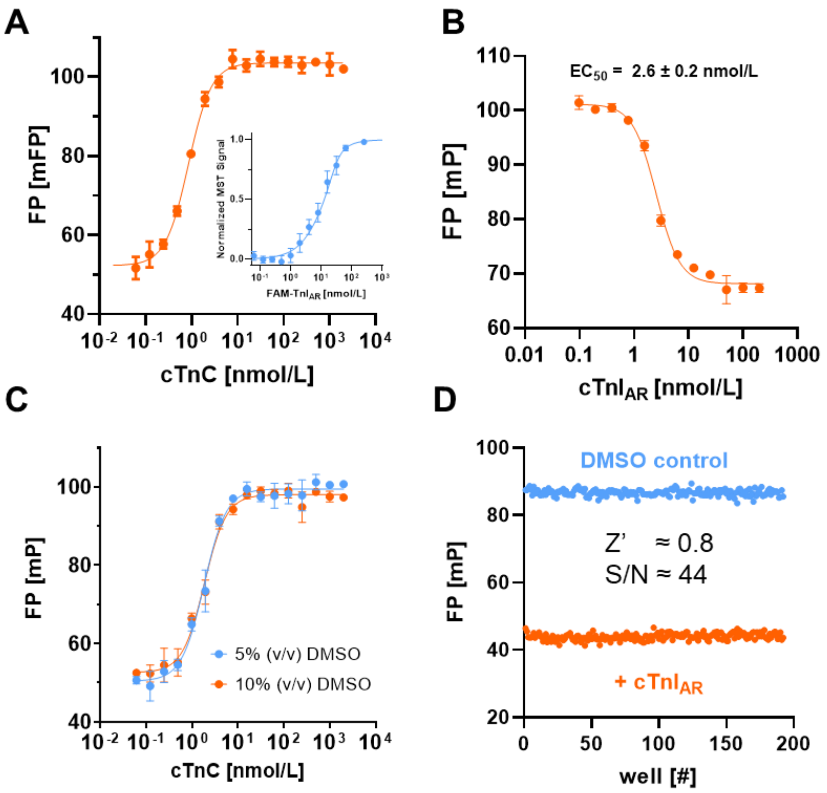

Figure S1. Characterization and Validation of the cTnI$_{AR}$ HTS assay (A) Interaction of FAM-cTnI$_{AR}$ with cTnC monitored by fluorescence polarization (FP). FAM-cTnI$_{AR}$ was titrated against increasing concentrations of cTnC (orange). Inset: Interaction of Alexa647-labelled cTnC and FAM-cTnI$_{AR}$ measured by Microscale Thermophoresis (blue). (B) Competitive titration of a mixture of 2 nmol/L FAM-cTnI$_{AR}$ and 5 nmol/L cTnC against increasing concentrations of unlabelled cTnI$_{AR}$ peptide monitored by FP. (C) Interaction of FAM-cTnI$_{AR}$ with cTnC monitored by fluorescence polarization (FP) in the presence of 5% (v/v) (blue) and 10% (v/v) orange dimethyl sulfoxide (DMSO). (D) FP measured from a mixture of FAM- cTnI$_{AR}$ and cTnC in the presence of either vehicle control (DMSO, blue) or 50 nmol/L unlabelled cTnI$_{AR}$ peptide (orange) in 384-well format. Means SEM, n=3-5.

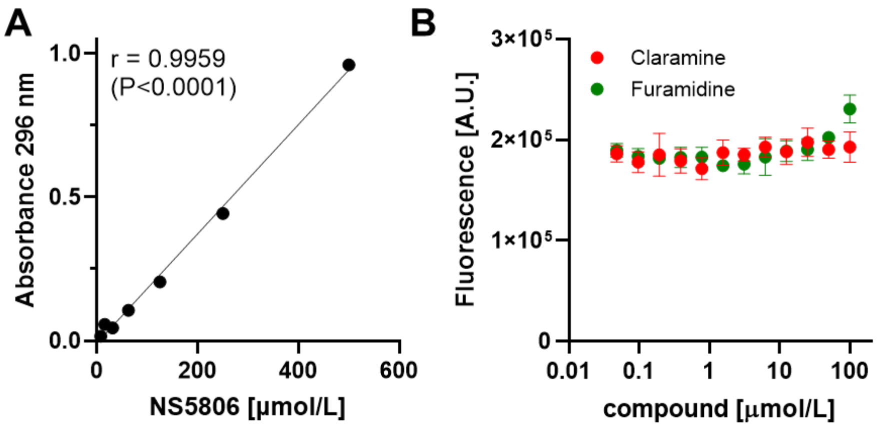

Figure S2. (A) Water solubility of NS5806. DMSO stocks of NS5806 were diluted with assay buffer to the indicated concentrations (fixed DMSO concentration of 0.1% (v/v)), potential aggregates removed by ultra-centrifugation and the absorbance at 296 nm plotted against the compound concentrations. The linear increase in absorbance suggests that NS5806 is soluble in aqueous buffers up to a concentration of at least 500 μmol/L. (B) Total fluorescent intensity dose-response curves for Claramine (red) and Furamidine (green) in the presence of 2 nmol/L FAM-cTnI$_{AR}$ and 5 nmol/L cTnC.

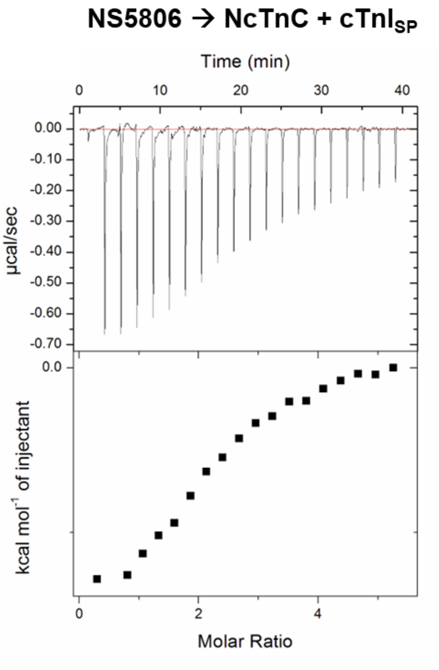

Figure S3. Thermogram of NS5806 titration into a mixture of the Ca^{2+}-saturated N-lobe of cTnC (NcTnC) and the cTnI switch peptide (SP).

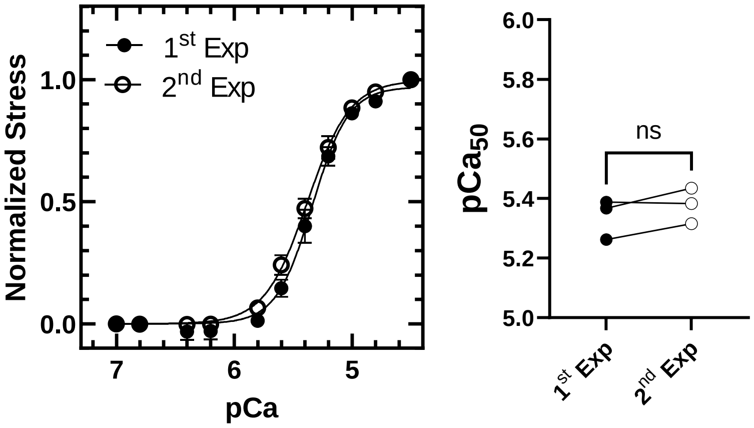

Figure S4. Force-pCa relation of demembranated human donor heart muscle strips for two consecutive experiments on the same preparation. Means ± SEM, n=3. Statistical significance of differences was assessed with a two-tailed student's t-test: ns-not significant.

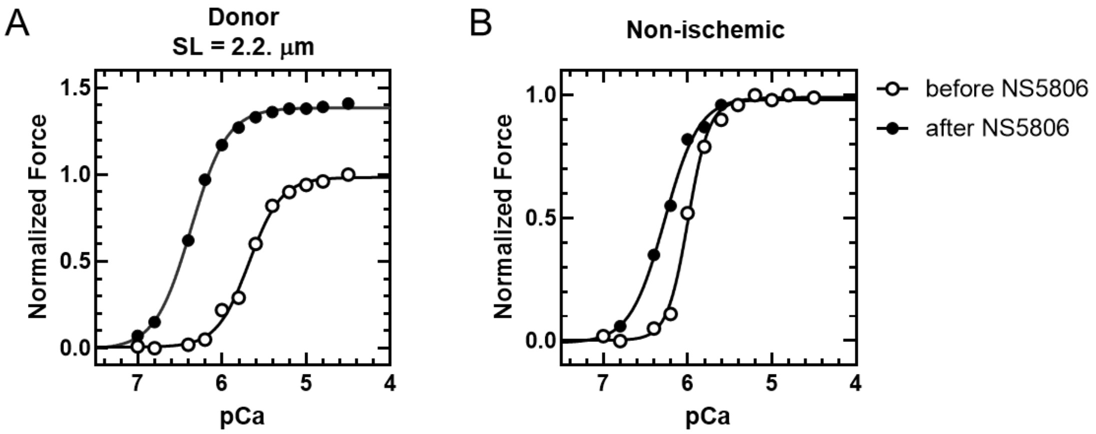

Figure S5. (A) Force-pCa relation of demembranated human donor heart muscle strips before (open circles) and after NS5806 incubation (closed circles) at long sarcomere length (2.2 μm). (B) Force-pCa relation of demembranated human heart muscle strips from ischemic heart failure patients before and after NS5806 incubation.

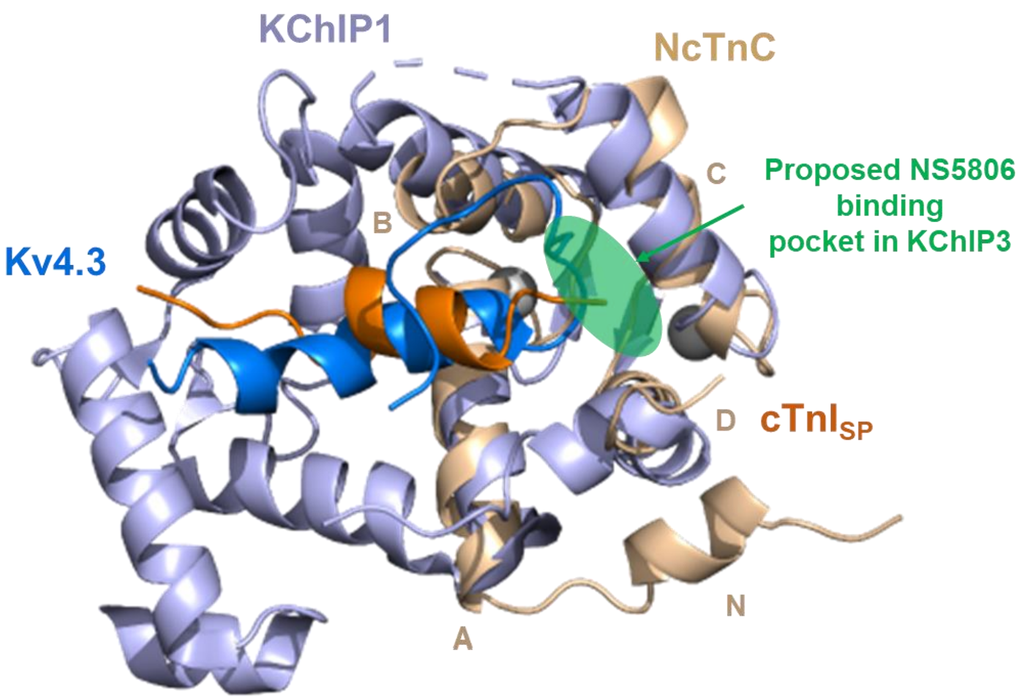

Figure S6. Structural alignment of the N-lobe of cTnC (NcTnC, yellow) bound to cTnI switch peptide ($cTnI_{SP}$, orange) (PDB entry 1MXL) with KChIP3 (purple) bound to a fragment of Kv4.3 (blue) (PDB entry 2I2R). NcTnC helices and proposed NS5806 binding site in KChIP are labelled accordingly.

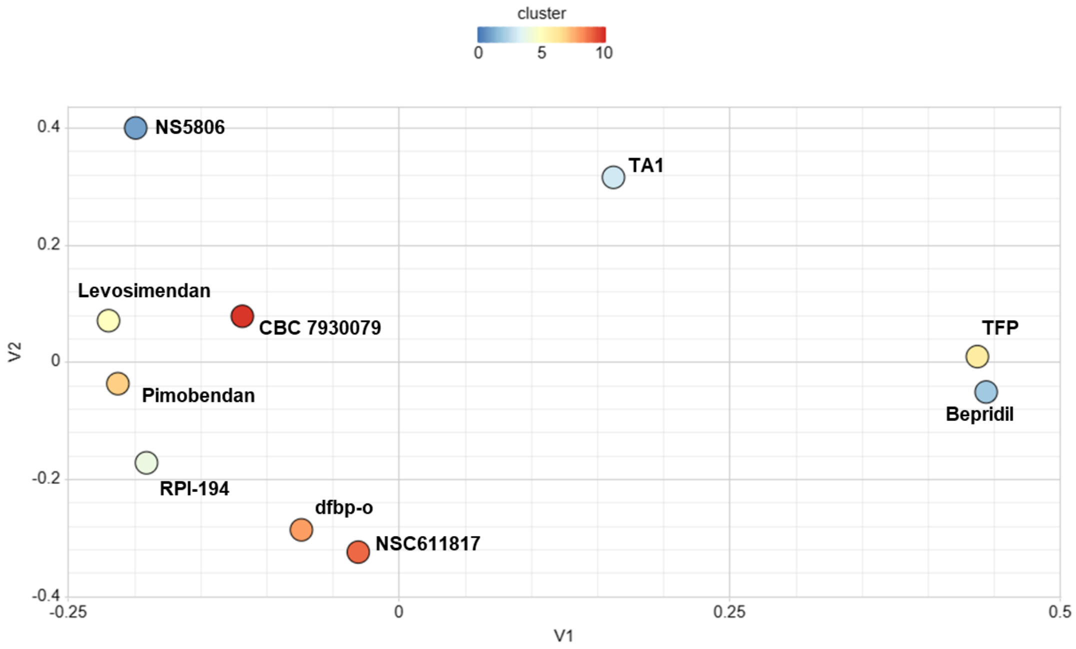

Figure S7. Structural clustering of known cardiac troponin C calcium sensitizers using multi-dimensional scaling (TFP – Trifluoperazine, CBC 7930079 – ChemBridge Compound 7930079).

Supplementary Information Tables and Table Legends

Table S1. Hit compounds identified in $cTnI_{SP}$ primary screens.

Library	Compound	FP (mP)
**ENZO Screenwell®**	Sertaconazole	88.2
	Clomiphene Citrate	63.2
	Gefitinib	83.7
	Chlorpromazine.HCl	88.9
	Fluphenazine.HCl	76.1
	Raloxifene.HCl	76.9
	Cinacalcet.HCl	82.4
	Fingolimod	66.1
	Clofazimine	85.2
	Toremifene Base	60.7
	Colistin Sulfate	78.5
	Thioridazine.HCl	74.3
	Tamoxifen Citrate	69.1
	Reserpine	88.4
	Sulconazole Nitrate	87.6
	Perphenazine	73.2
	Levothyroxine·Na	119.8
	Micafungin	128.6
**TOCRIScreen**	Tamoxifen citrate	62.2
	Raloxifene hydrochloride	87.9
	Raloxifene hydrochloride	82.1
	(S)-(+)-Niguldipine hydrochloride	85.7
	SR 33805 oxalate	79
	Tetrindole mesylate	65
	JTC 801	84.3
	Bax channel blocker	77.1
	NSC 23766	89.5
	PD 166285 dihydrochloride	78.7
	Dynole 34-2	64.1
	Y 134	78.9
	C 021 dihydrochloride	87.1
	NPS 2143 hydrochloride	87.5
	Bepridil hydrochloride	87
	GF 109203X	82.4
	PF 477736	64
	CCT 137690	83.6
	Ro 31-8220 mesylate	57.9
	Ro 3280	81.8
	IKK 16	82
	10-DEBC hydrochloride	82.8
	PHA 665752	76.4
	CASIN	80.4
	PQ 401	80.2
	DDR1-IN-1 dihydrochloride	74.4
	BMS 599626 dihydrochloride	88.2

	Mps1-IN-1 dihydrochloride	84.6
	GSK 1562590 hydrochloride	89.9
	TC-S 7003	73.5
	FRAX 486	83.2
	HKI 357	73.4
	AZ 191	83.4
	Sunitinib malate	83.4
	NVP ADW 742	68.4
	AMG 548	90
	XMD 8-87	81.2
	AEE 788	77.8
	CHR 6494 trifluoroacetate	81.9
	ERK5-IN-1	64.9
	TAK 960 hydrochloride	88
	SU 11274	64.5
	BS 181 dihydrochloride	73.2
	HTH 01-015	87.1
	ZM 447439	89.2
	Iressa	81.8
	UNC 1999	80.5
	Furamidine dihydrochloride	83.1
	AZ 6102	83.4
	WH-4-023	56.3
	Prochlorperazine dimaleate	78.2
	FTY 720	71.6
	Deltarasin	87.7
	DG 172 dihydrochloride	81
	Bazedoxifene acetate	74.5
	SP 100030	70.3
	Aripiprazole	86
	Calpeptin	121.8
	CFTRinh 172	127.9
	NS 3623	121.6
	NS 5806	137.6
**LOPAC®1280**	Chlorpromazine hydrochloride	91.3
	IKK-16 dihydrochloride	84.3
	Calmidazolium chloride	83.6
	Benazoline oxalate	83.7
	TNP	90.9
	Arbidol hydrochloride	89.4
	WZ4003	86.4
	Enclomiphene hydrochloride	78.4
	CYM50358	84.1
	Fluspirilene	88.6
	cis-(Z)-Flupenthixol dihydrochloride	87.6
	Ellipticine	86.1
	Fluphenazine dihydrochloride	87.3
	CID2858522	89.6
	Methiothepin mesylate	86.5
	NNC 55-0396	88.7
	AZ191	88.5

BMS-189453	88.8
Raloxifene hydrochloride	85.5
Pyridostatin trifluoroacetate salt	85.6
Tegaserod maleate	78.4
SID 3712249	86.6
Ruthenium red	83.7
Trifluoperazine dihydrochloride	84.5
Lasofoxifene tartrate	86.8
CCT137690	87
Thioridazine hydrochloride	89.4
HTH-01-015	87
Furamidine dihydrochloride	91.8
Prochlorperazine dimaleate	86.2
Palmitoyl-DL-Carnitine chloride	109.8
5HPP-33	112

Table S2. Hit compounds identified in $cTnI_{AR}$ primary screens.

Library	Compound	FP (mP)
**ENZO Screenwell®**	Carboplatin	67.3
	Oxcarbazepine	70.9
	Cisplatin (Cis-Diamineplatinum(Ii) Dichloride)	61.9
	Colistin Sulfate	71.7
	Chlorhexidine Dihydrochloride	58.8
	Indapamide	116.5
	Fomepizole	118
**TOCRIScreen**	WIN 64338 hydrochloride	71
	Furamidine dihydrochloride	73.2
	GSK 269962	104.8
	A 425619	105.5
	DQP 1105	103
	GF 109203X	104.5
	TC-S 7010	108.3
	AZD 3988	115.5
	Repaglinide	103.2
**LOPAC®1280**	Icaritin	64.6
	KT203	71.7
	Claramine	53.9
	SP600125	65
	Cisplatin	56.6
	Mitoxantrone	57.1
	Ruthenium red	44.3
	cDPCP	67.5
	GW2974	117.8
	Leukadherin-1	121.9
	U-74389G maleate	115.3
	Tyrphostin AG 112	115.8

Table S3. Summary of steady-state dissociation constants (K_d in μmol/L) of hit compounds binding to cardiac troponin constructs measured by isothermal titration calorimetry.

	**$Ca^{2+/}$NcTnC**	**Ca^{2+}/cChimera**	**cChimera**	**Ca^{2+}/NcTnC/$cTnI_{SP}$**	**Ca^{2+}/cTnC**
**NS5806**	60.7	7.6 ± 0.3	cnd	18.7	
**Furamidine**					11.9 ± 5.5
**Claramine**					21.6 ± 1.8

Means ± SEM, n=1-5. cnd-cannot be reliably determined.
